# Anti-inflammatory and Antioxidant Properties of Finger Millet (*Eleusine coracana* (L.) Gaertn.) Varieties Cultivated in Sri Lanka

**DOI:** 10.1155/2021/7744961

**Published:** 2021-10-01

**Authors:** Sooriya Arachchige Sachini Jayawardana, Jayanetti Koralalage Ramani Radhika Samarasekera, Gardhi Hettiarachchige Chamari Madhu Hettiarachchi, Jaanaki Gooneratne, Muhammad Iqbal Choudhary, Almas Jabeen

**Affiliations:** ^1^Industrial Technology Institute, No. 363, Bauddhaloka Mawatha, Colombo 07, Sri Lanka; ^2^Department of Chemistry, Faculty of Science, University of Colombo, Colombo 03, Sri Lanka; ^3^H. E. J. Research Institute of Chemistry, International Center for Chemical and Biological Sciences, University of Karachi, Karachi, Pakistan; ^4^Dr. Panjwani Center for Molecular Medicine and Drug Research, International Center for Chemical and Biological Sciences, University of Karachi, Karachi, Pakistan

## Abstract

The prevalence of inflammatory-mediated and oxidative stress-associated diseases is increasing worldwide, creating an increasing demand for novel sources of anti-inflammatory agents and antioxidants. This study was focused on determining the *in vitro* arachidonate 5-lipoxygenase (A5-LOX), xanthine oxidase (XO), hyaluronidase and oxidative burst inhibitory activities, and antioxidant properties of Ravi, Rawana, and Oshadha finger millet varieties using ethanolic and methanolic extracts. Among all extracts, the methanolic extract of Oshadha exhibited the highest A5-LOX (IC_50_ value: 484.42 *μ*g/ml) and XO (IC_50_ value: 764.34 *μ*g/ml) inhibitory activities. All extracts showed less than 50% hyaluronidase inhibitory activity at 1 mg/ml concentration. Methanolic extracts showed moderate inhibitory potential on reactive oxygen species (ROS) generated from whole blood phagocytes, with IC_50_ values ranging between 26.9 and 27.7 *μ*g/ml, when compared to ibuprofen (IC_50_ value: 11.18 *μ*g/ml). All extracts showed potent inhibition of ROS produced from polymorphonuclear neutrophils isolated from human blood when compared to ibuprofen (IC_50_ value: 2.47 *μ*g/ml) and IC_50_ values of methanolic and ethanolic extracts ranged from 0.29 to 0.47 *μ*g/ml and 1.35 to 1.70 *μ*g/ml, respectively. All extracts had significantly high amounts of phenolic compounds including flavonoids and the potential to scavenge 2,2′-azino-bis (3-ethylbenzothiazoline-6-sulfonic) acid (ABTS) cation, 2,2-diphenyl-1-picryl-hydrazyl (DPPH), and oxygen radicals. Besides, they were able to reduce metal ions and chelate metal ions terminating radical generating reactions. This is the first report of A5-LOX, XO, hyaluronidase, and oxidative burst inhibitory properties of any extract of any finger millet variety cultivated in Sri Lanka. The findings revealed the potential of using these finger millet extracts as natural sources of anti-inflammatory drug candidates. Additionally, the findings indicated that Ravi, Rawana, and Oshadha varieties are good sources of antioxidants. Therefore, consumption of these finger millet varieties on a regular basis may play an important role in the prevention and dietary management of oxidative stress-associated diseases.

## 1. Introduction

Inflammation is a defensive reaction of the immune system to eliminate harmful stimuli such as pathogens, damaged cells, and allergic or chemical irritations as well as to commence the healing process. Although inflammation is a normal response, if uncontrolled, excessive inflammation leads to many acute and chronic diseases. Currently, the prevalence of inflammatory-mediated diseases is increasing worldwide, creating an increasing demand for novel and effective anti-inflammatory agents [1, 2].

Arachidonate 5-lipoxygenase (A5-LOX) is a key enzyme which involves in the biosynthesis of potent mediators of inflammatory disorders and allergic reactions. It catalyzes the formation of leukotrienes from arachidonic acid [3]. Leukotrienes are endogenous mediators in the pathogenesis of a number of inflammatory diseases including asthma, allergic rhinitis, chronic bronchitis, rheumatoid arthritis, inflammatory bowel disease, and allergic reactions [3, 4]. Therefore, inhibition of A5-LOX is important in the prevention and treatment of various inflammatory disorders.

Xanthine oxidase (XO) catalyzes the oxidation of hypoxanthine to xanthine and xanthine to uric acid [5]. XO inhibitors block the biosynthesis of uric acid and consequently reduce the levels of uric acid as well as vascular oxidative stress. During the catalytic action of XO, reactive oxygen species (ROS) are formed, and therefore, XO plays a pathogenetic role in other inflammatory disorders as well. Consequently, XO inhibitors are important in the treatment of various inflammatory disorders accompanied by the catalytic action of XO [2, 5].

Hyaluronidase is a lysosomal enzyme which catalyzes the hydrolysis of mucopolysaccharides such as hyaluronic acid. In rheumatoid arthritis, hyaluronidase excessively degrades hyaluronic acid and reduces its amount and molecular weight, producing arthritic symptoms [6]. Inhibition of hyaluronic acid degradation is critical and imperative in controlling hyaluronidase-mediated pathological conditions [7].

In inflammatory conditions, immune cells are attracted to the site of inflammation. During defense and immunological reactions, NADPH oxidases residing in the immune cells are activated and generate ROS in large quantities creating an oxidative burst [1, 8]. Low and moderate quantities of ROS are beneficial in different physiological processes. However, overproduction of ROS deregulates the cellular functions which in turn enhances the inflammatory condition [8, 9]. Therefore, inhibition of ROS-induced oxidative burst is a potential remedial to prevent and manage inflammatory-mediated diseases.

During oxidative phosphorylation, free radicals including ROS are formed as by-products [10]. In addition to normal cellular metabolism, there are many other factors which lead the formation of free radicals, and if the rate of generating free radicals exceeds the rate of neutralization, oxidative stress is created, and it significantly contributes to all inflammatory diseases. Since antioxidants are capable of preventing the formation of free radicals and quenching free radicals, a balance between antioxidants and free radicals is essential to maintain the physiological functions properly [8, 11, 12].

Finger millet (*Eleusine coracana* (L.) Gaertn.) is the most important small millet in the tropics, and several therapeutic properties of finger millet have been reported previously [13–17]. However, for the finger millet varieties which are currently cultivated and consumed in Sri Lanka, there is a lack of scientific evidences on the therapeutic properties and potential health benefits to the consumers. Consequently, it is imperative to study the anti-inflammatory and antioxidant properties of Sri Lankan finger millet varieties and to recognize their potential to improve the nutritional and health status of the consumers. The prevalence of inflammatory-mediated and oxidative stress-associated diseases is increasing worldwide, creating an emerging demand for novel sources of anti-inflammatory agents and antioxidants [1, 2]. With this background, the present study was focused on evaluating Sri Lankan finger millet varieties for *in vitro* anti-inflammatory properties in terms of A5-LOX, XO, hyaluronidase, and oxidative burst inhibitory activities and antioxidant properties using different antioxidant assays.

## 2. Materials and Methods

### 2.1. Sample Collection and Preparation

Finger millet varieties which are recommended for cultivation in Sri Lanka by the Department of Agriculture, namely, Ravi, Rawana, and Oshadha, were collected from Field Crops Research and Development Institute (FCRDI), Mahailuppallama, Sri Lanka. These varieties were cultivated in experimental plots in the Low Country Dry Zone, at FCRDI, Mahailuppallama, and the seeds were certified by the Seed Certification Service of Department of Agriculture, Sri Lanka.

Finger millet seeds were dehulled (TM 05C, Satake Corporation, Japan), and flours from whole grains were obtained by milling (Pulverisette 14, Fritsch, Germany) and passing through a 0.5 mm sieve. Flours of whole grains were extracted with ethanol and methanol separately. Whole grain flour (100 g) was extracted with the solvent (400 ml) for overnight at room temperature (28 ± 2°C) using a magnetic stirrer and centrifuged at 4000 rpm for 20 min. The supernatant was collected separately, and the residue was reextracted twice using the same conditions. The supernatants were combined and evaporated to dryness under reduced pressure at 40°C using a rotary evaporator (R-114, Büchi Labortechnik AG, Switzerland). The solvent-free extracts were stored in airtight glass containers at -20°C until use for the analysis.

### 2.2. Enzymes, Chemicals, and Equipment

Folin-Ciocalteu phenol reagent, aluminium chloride, 2,4,6-Tri(2-pyridyl)-s-triazine (TPTZ), 2,2′-azobis (2-amidinopropane) dihydrochloride (AAPH), 2,2-diphenyl-1-picrylhydrazyl (DPPH), quercetin, 6-hydroxy-2,5,7,8-tetramethylchroman-2-carboxylic acid (trolox), ethylenediaminetetraacetic acid (EDTA), 2,2′-azino-bis(3-ethylbenzothiazoline-6-sulfonic acid) diammonium salt (ABTS), fluorecein, 3-(2-Pyridyl)-5,6-diphenyl-1,2,4-triazine-*p*,*p*′-disulfonic acid monosodium salt hydrate (ferrozine), arachidonate 5-lipoxygenase, linoleic acid, baicalein, xanthine oxidase, xanthine, allopurinol, hyaluronidase, hyaluronic acid sodium salt, p-dimethylaminobenzaldehyde, gallic acid, tannic acid, dimethyl sulfoxide, and ibuprofen were purchased from Sigma-Aldrich, MO, USA. HBSS^++^ and HBSS^--^ were purchased from Thermo Fisher Scientific Inc., Massachusetts, USA. Zymosan was purchased from Wako Pure Chemical Industries Ltd., Osaka, Japan. Luminol (3-aminophthalhydrazide) was purchased from Alfa Aesar GmbH & Co KG, Karlsruhe, Germany. Lymphocyte separation medium was purchased from MP Biomedicals, Inc., Ohio, USA. All other chemicals and reagents used in the experiments were of ACS, HPLC, and analytical grades. To determine the oxygen radical absorbance capacity, a fluorescence microplate reader (SpectraMax–Gemini EM, Molecular Devices Inc., USA) was used. For chemiluminescence assays, a luminometer (Luminoskan RS, Labsystems, Finland) was used. For all other bioassays, a microplate reader (SpectraMax Plus^384^, Molecular Devices Inc., USA) was used.

### 2.3. Determination of Total Phenolic Content

Total phenolic content (TPC) was determined according to the Folin-Ciocalteu method [18]. Finger millet extract (20 *μ*l) was mixed with 110 *μ*l of freshly prepared 10 times diluted Folin-Ciocalteu reagent and 70 *μ*l of sodium carbonate solution (10% *w*/*v*) and incubated at room temperature (RT) for 30 min. Absorbance was measured at 765 nm. Gallic acid was used to plot the standard curve (*y* = 0.0532*x* + 0.0339; *r*^2^ = 0.9992), and TPC was calculated as mg gallic acid equivalents (GAE) per 100 g of flour on a dry weight basis.

### 2.4. Determination of Total Flavonoid Content

Total flavonoid content (TFC) was determined according to the aluminium chloride colorimetric method [19]. Finger millet extract (100 *μ*l) was mixed with aluminium chloride solution (2% *w*/*v*, 100 *μ*l), incubated for 10 min at RT, and the absorbance was measured at 415 nm. Quercetin was used to plot the standard curve (*y* = 0.0349*x* − 0.2091; *r*^2^ = 0.9974), and TFC was calculated as mg quercetin equivalents per 100 g of flour on a dry weight basis.

### 2.5. Determination of DPPH Radical Scavenging Activity

The ability to scavenge DPPH radicals was determined according to the method described by Blois [20]. Finger millet extract (50 *μ*l) was mixed with 90 *μ*l of methanol and 60 *μ*l of DPPH solution (0.02% *w*/*v*) and incubated at RT in the dark for 10 min. Absorbance values of the sample (As) and control (Ac) were measured at 517 nm. Trolox was used as the standard. DPPH radical scavenging activity as percentage inhibition was calculated using Equation (1). IC_50_ value was calculated using dose-response graphs.

(1) $$\begin{array}{c} \text{Inhibition }\left(\%\right)=\left[\frac{\left(\text{Ac}-\text{As}\right)}{\text{Ac}}\right]\times 100. \end{array}$$

### 2.6. Determination of ABTS Cation Radical Scavenging Activity

The ability to scavenge ABTS cation radicals was determined according to the method described by Re et al. [21] with some modifications. ABTS cation radical solution was prepared by incubating 10 mg of ABTS in 2.5 ml of potassium persulphate at 37°C in the dark for 16 h and diluting 7 times with 50 mM phosphate buffer saline (pH 7.4). Finger millet extract (12.5 *μ*l) was mixed with phosphate buffer saline (147.5 *μ*l) and freshly prepared ABTS cation radical solution (40 *μ*l) and incubated at RT in the dark for 10 min. Absorbance values of the sample (As) and control (Ac) were measured at 734 nm. Trolox was used as the standard. ABTS cation radical scavenging activity as percentage inhibition was calculated using Equation (2). IC_50_ value was calculated using dose-response graphs.

(2) $$\begin{array}{c} \text{Inhibition }\left(\%\right)=\left[\frac{\left(\text{Ac}-\text{As}\right)}{\text{Ac}}\right]\times 100. \end{array}$$

### 2.7. Determination of Oxygen Radical Absorbance Capacity (ORAC)

ORAC was evaluated according to the method described by Ou et al. [22] with some modifications. Fluorescein (4.8 *μ*M) and AAPH (40 mg/ml) solutions were prepared in 75 mM phosphate buffer (pH 7.4). Finger millet extract (10 *μ*l) was mixed with 40 *μ*l of phosphate buffer and 100 *μ*l of fluorescein and incubated at 37°C for 10 min. AAPH (50 *μ*l) was added, and the decay of fluorescein was measured at excitation and emission wavelengths of 494 nm and 535 nm, respectively, at 37°C for 35 min at 1 min intervals using a fluorescence microplate reader. Trolox was used as the standard. Area under the curve (AUC) values of the sample (AUC_*S*_), control (AUC_*C*_), and trolox (AUC_*T*_) were recorded. ORAC value was calculated using Equation (3) where conc. stands for concentration. The results were expressed as mg trolox equivalents per 100 g of flour on a dry weight basis.

(3) $$\begin{array}{c} \text{ORAC value}=\left[\frac{\left(\text{AUCS}-\text{AUCC}\right)}{\text{AUCT}-\text{AUCC}}\right]\times \left[\frac{\left(\text{Trolox conc}.\right)}{\text{Sample conc}.}\right]. \end{array}$$

### 2.8. Determination of Ferrous Ion Chelating (FIC) Activity

The ability to chelate ferrous ions was determined according to the method described by Carter [23]. Finger millet extract (100 *μ*l) was mixed with 1 mM ferrous sulphate solution (20 *μ*l) and 40 *μ*l of distilled water. Then, 1 mM ferrozine solution (40 *μ*l) was added and incubated for 10 min at RT. Absorbance values of the sample (As) and control (Ac) were recorded at 562 nm. EDTA was used as the standard. FIC activity as percentage chelation was calculated using Equation (4).

(4) $$\begin{array}{c} \text{Chelation }\left(\%\right)=\left[\frac{\left(\text{Ac}-\text{As}\right)}{\text{Ac}}\right]\times 100. \end{array}$$

### 2.9. Determination of Ferric Reducing Antioxidant Power (FRAP)

FRAP was determined according to the method described by Benzine and Szeto [24] with some modifications. FRAP reagent was prepared by mixing 300 mM acetate buffer (pH 3.6), 20 mM ferric chloride, and 10 mM TPTZ in a ratio of 10:1:1 and incubating at 37°C for 10 min. Finger millet extract (20 *μ*l) was mixed with 30 *μ*l of acetate buffer and 150 *μ*l of freshly prepared FRAP reagent, incubated at RT for 8 min, and absorbance was recorded at 600 nm. Trolox was used to plot the standard curve (*y* = 0.17*x* + 0.15; *r*^2^ = 1.00), and FRAP was calculated as mg trolox equivalents per 100 g of flour on a dry weight basis.

### 2.10. Determination of A5-LOX Inhibitory Activity

A5-LOX inhibitory activity was evaluated according to the method described by Tappel [25] with some modifications. Finger millet extract (10 *μ*l) was mixed with 115 *μ*l of 100 mM sodium phosphate buffer (pH 8.0) and 50 *μ*l of A5-LOX solution (5000 U/ml) and incubated at RT for 10 min. The reaction was initiated with the addition of 25 *μ*l of 0.08 mM linoleic acid. The change of absorbance was recorded at 234 nm at 1 min intervals for 10 min at RT, and the maximum velocity (*V*_max_) values of the sample (*V*_max*S*_) and control (*V*_max*C*_) were recorded. Baicalein was used as the standard. A5-LOX inhibitory activity as percentage inhibition was calculated using Equation (5). IC_50_ value was calculated using dose-response graphs.

(5) $$\begin{array}{c} \text{Inhibition }\left(\%\right)=\left[\frac{\left(V_{\max C}-V_{\max S}\right)}{V_{\max C}}\right]\times 100. \end{array}$$

### 2.11. Determination of XO Inhibitory Activity

XO inhibitory activity was determined according to the method described by Lee et al. [26] with some modifications. Finger millet extract (10 *μ*l) was mixed with 150 *μ*l of 50 mM sodium phosphate buffer (pH 7.4) and 20 *μ*l of XO (0.15 U/ml) and incubated for 10 min at RT. The reaction was initiated with the addition of 20 *μ*l of 0.1 mM xanthine. The change of absorbance was recorded at 295 nm at 1 min intervals for 15 min at RT, and *V*_max_ values of the sample (*V*_max*S*_) and control (*V*_max*C*_) were recorded. Allopurinol was used as the standard. XO inhibitory activity as percentage inhibition was calculated using Equation (6). IC_50_ value was calculated using dose-response graphs.

(6) $$\begin{array}{c} \text{Inhibition }\left(\%\right)=\left[\frac{\left(V_{\max C}-V_{\max S}\right)}{V_{\max C}}\right]\times 100. \end{array}$$

### 2.12. Determination of Hyaluronidase Inhibitory Activity

Hyaluronidase inhibitory activity was evaluated according to the method described by Sahasrabudhe and Dedhar [27] with some modifications. Finger millet extract (50 *μ*l) was mixed with 10 *μ*l of hyaluronidase (8400 U/ml) and incubated at 37°C for 10 min. The enzyme was activated by adding 20 *μ*l of calcium chloride (12.5 mM) and incubating at 37°C for 10 min. The reaction was initiated with the addition of sodium hyaluronate (50 *μ*l) and incubated at 37°C for 40 min. Then, 10 *μ*l of 0.9 M sodium hydroxide and 20 *μ*l of 0.2 M sodium borate were added and incubated at 100°C for 3 min. After cooling to RT, 50 *μ*l of 67 mM p-dimethylaminobenzaldehyde was added and incubated at 37°C for 10 min. Absorbance values of the sample (As) and control (Ac) were measured at 585 nm. Tannic acid was used as the standard. Hyaluronidase inhibitory activity as percentage inhibition was calculated using Equation (7).

(7) $$\begin{array}{c} \text{Inhibition }\left(\%\right)=\left[\frac{\left(\text{Ac}-\text{As}\right)}{\text{Ac}}\right]\times 100. \end{array}$$

### 2.13. Determination of Oxidative Burst Inhibitory Activities

Anti-inflammatory potentials in terms of oxidative burst inhibitory activities in whole human blood and isolated polymorphonuclear neutrophils were determined using the luminol enhanced chemiluminescence assay [28, 29] with some modifications. Experiments were conducted at Dr. Panjwani Center for Molecular Medicine and Drug Research, International Center for Chemical and Biological Sciences, University of Karachi, Pakistan, and the institute has the ethical clearance for studies on human blood from the independent ethics committee, ICCBS, UoK. No: ICCBS/IEC-008-BC-2015/Protocol/1.0.

#### 2.13.1. Determination of Oxidative Burst Inhibitory Activity in Whole Human Blood

Human blood (1 ml) was aseptically collected by vein puncture into a heparinized tube from a healthy nonsmoking volunteer, who did not take any medication or supplements for more than one week, and diluted (1:20 dilution) with HBSS^++^ (Hanks Balanced Salt Solution, containing calcium and magnesium). Finger millet extract (25 *μ*l) was mixed with diluted whole blood (25 *μ*l) and incubated at 37°C for 15 min. Then, 25 *μ*l of serum opsonized zymosan and 25 *μ*l of luminol were added. The oxidative burst ROS production was monitored for 50 min using a luminometer with repeated scans at 30 s intervals and 1 s points measuring time. Ibuprofen was used as the standard drug. Inhibition percentage was calculated using Equation (8).

(8) $$\begin{array}{c} \text{Inhibition }\left(\%\right)=\left[\frac{{\text{RLU}}_{C}-{\text{RLU}}_{S}}{\text{RLUC}}\right]\times 100, \end{array}$$

where RLU_*C*_ is the luminometer reading in terms of relative light units (RLU) for control and RLU_*S*_ is the luminometer reading in RLU for the sample. IC_50_ value was calculated using dose-response graphs.

#### 2.13.2. Determination of Oxidative Burst Inhibitory Activity in Isolated Polymorphonuclear Neutrophils

Whole human blood (10 ml) was mixed with equal volumes of HBSS^--^ (Hanks Balanced Salt Solution, without calcium and magnesium) and lymphocyte separation medium (LSM) and allowed erythrocytes to settle down for 30 min at RT. Then, the plasma was laid carefully on LSM and centrifuged at 400 × g for 20 min at RT. Supernatant was removed; red blood cells in the resulting pellet were lysed by mixing with cold deionized water, mixed with cold HBSS^--^, and centrifuged at 300 × g for 10 min at 4°C. The pellet was washed twice, and the viability of the resulting polymorphonuclear neutrophils was checked using the Trypan blue exclusion method. The concentration of cells was adjusted to 1 × 10^6^ cells/ml. Isolated polymorphonuclear neutrophils (25 *μ*l) were mixed with finger millet extract (25 *μ*l) and incubated for 15 min at 37°C. Then, 25 *μ*l of serum opsonized zymosan and 25 *μ*l of luminol were added, and oxidative burst ROS production was monitored for 50 min using a luminometer with repeated scans at 30 s intervals and 1 s points measuring time. Ibuprofen was used as the standard drug. Inhibition percentage was calculated using Equation (8).

### 2.14. Statistical Analysis

Data of each experiment were statistically analysed using the IBM SPSS Statistics (version 20) software, and the results were expressed as mean ± standard error (SE). Statistical significance was set at 95% confidence level. One way analysis of variance (ANOVA) and Tukey's test were used to determine the differences among the varieties and extracts. The Pearson's correlation coefficient was used for the correlation analysis.

## 3. Results and Discussion

### 3.1. Antioxidant Properties of Finger Millet Varieties

#### 3.1.1. Total Phenolic and Total Flavonoid Contents

TPC and TFC of ethanolic and methanolic extracts of Ravi, Rawana, and Oshadha finger millet varieties are presented in Table 1. There were significant differences (*P* < 0.05) among the varieties and between the extracts. Methanolic extracts had significantly high (*P* < 0.05) TPCs and TFCs compared to ethanolic extracts in all finger millet varieties. Among the three varieties, Oshadha had a significantly high (*P* < 0.05) TPC, and Ravi had a significantly high (*P* < 0.05) TFC. There were no significant differences (*P* ≥ 0.05) between the TFCs of Rawana and Oshadha.

Significant negative (*P* < 0.05) correlations were observed between TPCs and IC_50_ values of DPPH (*r* = −0.602) and ABTS cation (*r* = −0.836) radical scavenging activities of all extracts. In addition, a significant positive (*P* < 0.05) correlation was observed between TPCs and ORAC values (*r* = 0.787) of the extracts. These correlations indicated the involvement of finger millet phenols in radical scavenging activities. TPCs of the extracts positively correlated with FRAP values (*r* = 0.876, *P* < 0.05) and FIC activities (*r* = 0.473, *P* < 0.05) of the extracts indicating the ability of finger millet phenols to reduce ferric ions and chelate ferrous ions. There were significant negative (*P* < 0.05) correlations between TFCs and IC_50_ values of DPPH (*r* = −0.802) and ABTS cation (*r* = −0.471) radical scavenging activities of the extracts. Besides, TFCs positively correlated with FIC activities (*r* = 0.689, *P* < 0.05) of the extracts, suggesting the involvement of finger millet flavonoids in scavenging radicals and chelating ferrous ions.

TPCs of common cereals were reported by Abeysekera et al. [30]. According to their findings, TPCs of methanolic extracts of white and red rice varieties ranged from 29.34 to 139.79 mg GAEs per 100 g of flour on a dry weight basis, while TPCs of ethanolic extracts ranged from 59.78 to 113.56 mg GAEs per 100 g of flour on a dry weight basis. TPCs of methanolic extracts of barley, corn, wheat, and oats were 93.61, 75.10, 71.22, and 17.24 mg GAEs per 100 g of flour, respectively, on a dry weight basis. TPCs of ethanolic extracts of barley, corn, wheat, and oats were 68.69, 52.10, 78.05, and 15.72 mg GAEs per 100 g of flour, respectively, on a dry weight basis. Therefore, when compared with the aforementioned findings, Ravi, Rawana, and Oshadha finger millet varieties possess high phenolic contents than commonly consumed cereals such as rice, wheat, corn, barley, and oats. Phenolic compounds are known to be strong antioxidants, and therefore, any plant tissue possessing these compounds can be potentially used as effective natural antioxidants [2]. Consequently, the high phenolic contents indicated the antioxidant potential of Ravi, Rawana, and Oshadha varieties.

#### 3.1.2. DPPH and ABTS Cation Radical Scavenging Activities

Significant differences (*P* < 0.05) were observed in both DPPH and ABTS cation radical scavenging activities of Ravi, Rawana, and Oshadha varieties and between the extracts (Table 2). Methanolic extracts of the three varieties showed significantly high (*P* < 0.05) DPPH and ABTS cation radical scavenging activities when compared to ethanolic extracts. Ravi and Oshadha varieties showed significantly high (*P* < 0.05) DPPH and ABTS cation radical scavenging activities when compared to Rawana. All extracts showed dose-dependent DPPH and ABTS cation radical scavenging activities. In DPPH radical scavenging assay, the methanolic extract of Oshadha had the lowest IC_50_ value indicating the highest activity, and in ABTS cation radical scavenging assay, methanolic extract of Ravi had the lowest IC_50_ value indicating the highest activity.

Significant positive (*P* < 0.05) correlations were observed between IC_50_ values of DPPH and ABTS cation radical scavenging activities of the extracts (*r* = 0.750) indicating that the finger millet extracts had comparable activities in the two assays. The compounds present in finger millet extracts which can scavenge DPPH radicals may be capable of scavenging ABTS cation radicals as well. Almeida et al. [31] have observed a similar correlation when studying the antioxidant activities of Brazilian fruit extracts. Suriano et al. [32] also have reported a similar correlation in barley varieties, and they have attributed it to the same reaction mechanism on which both assays rely on. A good antioxidant should be capable of quenching free radicals [33, 34]. Therefore, the abilities to scavenge DPPH and ABTS cation radicals indicated the antioxidant potential of Ravi, Rawana, and Oshadha finger millet varieties.

#### 3.1.3. Oxygen Radical Absorbance Capacity

There were significant differences (*P* < 0.05) in scavenging oxygen radicals among the Ravi, Rawana, and Oshadha varieties (Table 3), and ORAC value of Oshadha was significantly higher (*P* < 0.05) than that of Ravi and Rawana varieties. In Rawana, the ethanolic extract showed significantly high (*P* < 0.05) ORAC value when compared to the methanolic extract. There were no significant differences (*P* ≥ 0.05) between the ORAC values of ethanolic and methanolic extracts in Ravi and Oshadha varieties.

ORAC values of common cereals were reported in previous studies [30]. ORAC values of methanolic extracts of white and red rice varieties ranged from 150.61 to 439.64 mg trolox equivalents per 100 g of flour on a dry weight basis, while ORAC values of ethanolic extracts ranged from 108.32 to 272.08 mg trolox equivalents per 100 g of flour on a dry weight basis. ORAC values of methanolic extracts of barley, wheat, corn, and oats were 357.51, 322.00, 249.91, and 50.71 mg trolox equivalents per 100 g of flour, respectively, on a dry weight basis. ORAC values of ethanolic extracts of barley, wheat, corn, and oats were 244.33, 263.90, 84.04, and 73.20 mg trolox equivalents per 100 g of flour, respectively, on a dry weight basis. Therefore, when compared with the aforementioned findings, Ravi, Rawana, and Oshadha finger millet varieties possess higher abilities to scavenge peroxyl radicals than the other commonly consumed cereals such as rice, wheat, corn, barley, and oats. Since ability of an extract to scavenge physiological radicals such as peroxyl radicals is an important indicator of its antioxidant capacity [33, 34], these findings indicated the antioxidant potential of Ravi, Rawana, and Oshadha varieties.

#### 3.1.4. Ferrous Ion Chelating Activity

There were significant differences (*P* < 0.05) in FIC activities of Ravi, Rawana, and Oshadha varieties and between the extracts (Table 3). Methanolic extracts showed significantly high (*P* < 0.05) activities when compared to ethanolic extracts of all finger millet varieties. Therefore, methanol extraction may be more efficient compared to ethanol extraction when extracting compounds, with FIC ability, from finger millet varieties. Ferrous ions can initiate and spread many radical generating reactions, and consequently, chelating ferrous ions are important to prevent redox active metal catalysis-associated ROS generating reactions [35]. Therefore, the abilities to chelate ferrous ions reflected the antioxidant potential of Ravi, Rawana, and Oshadha varieties.

#### 3.1.5. Ferric Reducing Antioxidant Power

There were significant differences (*P* < 0.05) in FRAP values of Ravi, Rawana, and Oshadha varieties and between the extracts (Table 3). Methanolic extracts showed significantly high (*P* < 0.05) FRAP values when compared to ethanolic extracts. Among the three varieties, Oshadha showed significantly high (*P* < 0.05) FRAP value when compared to Ravi and Rawana. Since the reducing capacity of an extract is an important indicator of its antioxidant capacity [2], these results provided evidences for the antioxidant potential of Ravi, Rawana, and Oshadha varieties.

FRAP values of methanolic extracts of white and red rice varieties ranged from 61.79 to 743.18 mg trolox equivalents per 100 g of flour on a dry weight basis, while FRAP values of ethanolic extracts ranged from 139.36 to 255.34 mg trolox equivalents per 100 g of flour on a dry weight basis. FRAP values of methanolic extracts of barley, wheat, corn, and oats were 199.45, 177.95, 81.06, and 61.74 mg trolox equivalents per 100 g of flour, respectively, on a dry weight basis. FRAP values of ethanolic extracts of barley, wheat, corn, and oats were 107.21, 84.27, 49.05, and 26.60 mg trolox equivalents per 100 g of flour, respectively, on a dry weight basis [30]. Therefore, when compared with the aforementioned findings, Ravi, Rawana, and Oshadha finger millet varieties possess higher abilities to reduce ferric ions when compared to rice, wheat, corn, barley, and oats. Since reducing capacity of an extract is an important indicator of its antioxidant capacity [2], the abilities to reduce ferric ions indicated the antioxidant potential of Ravi, Rawana, and Oshadha varieties.

### 3.2. Anti-inflammatory Properties of Finger Millet Varieties

#### 3.2.1. A5-LOX Enzyme Inhibitory Activity

A5-LOX enzyme inhibitory activities of the ethanolic and methanolic extracts of Ravi, Rawana, and Oshadha varieties are given in Table 4. Both extracts of the three finger millet varieties are capable of inhibiting the catalytic action of the A5-LOX enzyme in a dose-dependent manner. Methanolic extracts of the three varieties exhibited significantly (*P* < 0.05) high A5-LOX enzyme inhibitory activities when compared to ethanolic extracts. Among the six finger millet extracts, the methanolic extract of Oshadha had the lowest IC_50_ value indicating the highest A5-LOX enzyme inhibitory activity.

IC_50_ values of A5-LOX enzyme inhibitory activities of the extracts were negatively correlated with TPCs (*r* = −0.788, *P* < 0.05) and TFCs (*r* = −0.399, *P* < 0.05) of the extracts, indicating the involvement of finger millet phenols, including flavonoids, in inhibiting the catalytic action of A5-LOX enzyme. Perera et al. [2] have observed a similar correlation in Sri Lankan medicinal plants, and they have reported that anti-A5-LOX activity is proportional to the polyphenol and flavonoid contents. This is supported by the fact that most of A5-LOX enzyme inhibitors are phenols, and furthermore, the most potent A5-LOX enzyme inhibitors, including baicalein, are flavonoids [36].

There were strong positive correlations between IC_50_ values of A5-LOX enzyme inhibitory activities and IC_50_ values of DPPH (*r* = 0.840, *P* < 0.05) and ABTS cation (*r* = 0.660, *P* < 0.05) radical scavenging activities of all extracts. These correlations indicated the involvement of antioxidants, which are present in finger millet extracts, in inhibiting the A5-LOX enzyme by scavenging the radical intermediates which are generated during the catalytic action of A5-LOX enzyme. A nonheme iron atom is present in the active site of lipoxygenases. The catalytic reaction of lipoxygenases involves a single electron oxidation at the active site iron atom which switches between Fe^2+^ and Fe^3+^ redox states. Therefore, molecules with iron-chelating and ferric ion reducing functionalities can act as potent A5-LOX inhibitors [2, 37]. In the present study, also strong negative correlations were observed between the IC_50_ values of A5-LOX enzyme inhibitory activities and FRAP values (*r* = −0.873, *P* < 0.05) as well as FIC activities (*r* = −0.752, *P* < 0.05) of all extracts suggesting that reducing the ferric ions and chelating the ferrous ions as potential mechanisms of finger millet extracts in inhibiting the catalytic action of the A5-LOX enzyme.

#### 3.2.2. XO Enzyme Inhibitory Activity

Both ethanolic and methanolic extracts of Ravi, Rawana, and Oshadha finger millet varieties were capable of inhibiting the catalytic action of the xanthine oxidase enzyme in a dose-dependent manner indicating the anti-inflammatory potential of the three varieties (Table 4). Methanolic extracts exhibited significantly (*P* < 0.05) high XO enzyme inhibitory activities when compared to ethanolic extracts. Among the six finger millet extracts, methanolic extract of Oshadha had the lowest IC_50_ value indicating the highest XO enzyme inhibitory activity.

IC_50_ values of XO enzyme inhibitory activities of all extracts showed significant (*P* < 0.05) negative correlations with TPCs (*r* = −0.744), TFCs (*r* = −0.489), FRAP values (*r* = −0.793), and FIC activities (*r* = −0.906) indicating the involvement of antioxidants, which are present in finger millet extracts, in inhibiting the catalytic action of the enzyme. During the catalytic action of XO, superoxide anions and hydrogen peroxide are formed [38]. The significant (*P* < 0.05) positive correlations between IC_50_ values of XO enzyme inhibitory activities and IC_50_ values of DPPH (*r* = 0.826) and ABTS cation (*r* = 0.790) radical scavenging activities of all extracts suggested, scavenging radical intermediates including ROS, which are generated during the catalytic action of the enzyme, as a potential mechanism of XO enzyme inhibition.

#### 3.2.3. Hyaluronidase Enzyme Inhibitory Activity

**Figure 1:**
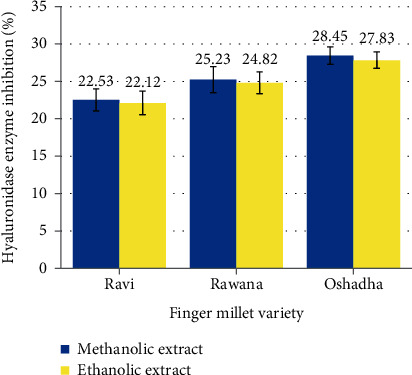
Hyaluronidase enzyme inhibitory activities of ethanolic and methanolic extracts of Sri Lankan finger millet varieties at 1 mg/ml assay concentration. Results are presented as mean values (*n* = 6), and the error bars represent the standard error.

Both ethanolic and methanolic extracts of Ravi, Rawana, and Oshadha finger millet varieties were capable of inhibiting the catalytic action of the hyaluronidase enzyme (Figure 1). Among the three varieties, Oshadha showed the highest hyaluronidase enzyme inhibitory activity although it was statistically not significant (*P* ≥ 0.05) when compared to the other two varieties. There were no significant differences (*P* ≥ 0.05) between the hyaluronidase enzyme inhibitory activities of the six extracts, and they were significantly (*P* < 0.05) lower than the hyaluronidase enzyme inhibitory activity of the reference standard, tannic acid which showed 99.16% inhibition at 0.5 mg/ml concentration. Girish et al. [7] and Sahasrabudhe and Deodhar [27] have reported that polyphenols are good hyaluronidase inhibitors. Furthermore, the reference standard used in this assay, tannic acid, is also a type of polyphenol [27]. In the present study also, hyaluronidase enzyme inhibitory activities of the extracts showed a positive correlation with TPCs (*r* = 0.387), confirming the involvement of finger millet phenols in inhibiting the catalytic action of hyaluronidase enzyme.

#### 3.2.4. Oxidative Burst Inhibitory Activity

The abilities of the ethanolic and methanolic extracts of Ravi, Rawana, and Oshadha finger millet varieties to inhibit the zymosan-induced oxidative burst in whole human blood were evaluated, and the results indicated that both extracts of the three finger millet varieties are capable of inhibiting oxidative burst in whole human blood in a dose-dependent manner (Table 4). Methanolic extracts exhibited significantly (*P* < 0.05) high inhibition when compared to ethanolic extracts. When comparing with ibuprofen, methanolic extracts had a moderate oxidative burst inhibitory potential in whole human blood. All extracts were further evaluated for their oxidative burst inhibitory activity in isolated polymorphonuclear neutrophils (Table 4). Similar to the results of the study with whole human blood, all extracts showed dose-dependent oxidative burst inhibitory activities in polymorphonuclear neutrophils, and methanolic extracts exhibited significantly (*P* < 0.05) high inhibition when compared to ethanolic extracts. According to the results of the present study, both ethanolic and methanolic extracts of finger millet varieties exhibited a high oxidative burst inhibition in isolated polymorphonuclear neutrophils when compared with ibuprofen. Methanolic extracts of Ravi, Rawana, and Oshadha exhibited 8.5, 5.2, and 5.6 times higher activities, respectively, for oxidative burst inhibition in human polymorphonuclear neutrophils when compared to ibuprofen. This may be attributed to the significantly high (*P* < 0.05) TPCs and TFCs (Table 1) of methanolic extracts of the three finger millet varieties.

TPCs of the extracts were found to be negatively correlated with IC_50_ values of oxidative burst inhibition in whole human blood (*r* = −0.431) and human polymorphonuclear neutrophils (*r* = −0.528, *P* < 0.05). Similarly, TFCs of the extracts were found to be negatively correlated with IC_50_ values of oxidative burst inhibition in whole human blood (*r* = −0.715, *P* < 0.05) and human polymorphonuclear neutrophils (*r* = −0.619, *P* < 0.05). These correlations indicated the involvement of finger millet phenols, including flavonoids, in oxidative burst inhibition. The significant (*P* < 0.05) negative correlations between FRAP values of the extracts and oxidative burst inhibitory activity in whole human blood (*r* = −0.541) and human polymorphonuclear neutrophils (*r* = −0.602) as well as FIC activities of the extracts and IC_50_ values of oxidative burst inhibition in whole human blood (*r* = −0.985) and human polymorphonuclear neutrophils (*r* = −0.959) suggested that antioxidant compounds which are present in the finger millet extracts are responsible for inhibiting the oxidative burst. There were significant (*P* < 0.05) positive correlations between IC_50_ values of ABTS cation radical scavenging activities and IC_50_ values of oxidative burst inhibitory activities in whole human blood (*r* = 0.556) and human polymorphonuclear neutrophils (*r* = 0.652). In addition, there were strong positive correlations between IC_50_ values of DPPH radical scavenging activities and IC_50_ values of oxidative burst inhibitory activities in whole human blood (*r* = 0.889, *P* < 0.05) and human polymorphonuclear neutrophils (*r* = 0.815, *P* < 0.05). These correlations indicated scavenging radicals as the prominent mechanism in oxidative burst inhibition. Antioxidants, which have a potential to inhibit ROS-induced oxidative burst, can serve as effective anti-inflammatory agents [1, 8]. Therefore, these results provided evidences for the anti-inflammatory potential of Ravi, Rawana, and Oshadha varieties. Oxidative burst inhibitory activities provided evidences for the anti-inflammatory potential of Ravi, Rawana, and Oshadha varieties while indicating the potential application of methanolic and ethanolic extracts of Ravi, Rawana, and Oshadha finger millet varieties in the prevention and management of ROS-induced inflammatory conditions as natural sources of anti-inflammatory drug candidates.

## 4. Conclusions

This is the first study revealing *in vitro* anti-inflammatory properties of any extract of any finger millet variety cultivated in Sri Lanka using A5-LOX, XO, hyaluronidase, and oxidative burst inhibitory assays. A5-LOX and xanthine oxidase enzyme inhibitory activities of Ravi, Rawana, and Oshadha finger millet varieties reflected the potential application in preventing various inflammatory disorders accompanied by the catalytic actions of A5-LOX and XO enzymes. Promising oxidative burst inhibitory activities in whole human blood and isolated polymorphonuclear neutrophils demonstrated that both ethanolic and methanolic extracts of Ravi, Rawana, and Oshadha finger millet varieties could be rich sources of oxidative burst inhibitory bioactive compounds and consequently reflected the potential application of Ravi, Rawana, and Oshadha finger millet varieties in prevention and management of ROS-induced inflammatory conditions. Owing to the findings on anti-inflammatory properties, Ravi, Rawana, and Oshadha finger millet varieties could be effectively used as natural sources of anti-inflammatory drug candidates, fulfilling the demand for novel anti-inflammatory agents which are derived from natural sources. Further cell-based studies will be conducted to verify these findings.

This study also revealed antioxidant properties of Ravi, Rawana, and Oshadha finger millet varieties. According to the findings, Ravi, Rawana, and Oshadha finger millet varieties contain significantly high amounts of phenolic compounds including flavonoids. All three finger millet varieties have the potential to scavenge free radicals including DPPH, ABTS cation, and oxygen radicals, to reduce metal ions and to chelate metal ions terminating radical generating reactions. These findings indicated that Ravi, Rawana, and Oshadha finger millet varieties could be good sources of antioxidants. Consequently, consumption of Ravi, Rawana, and Oshadha finger millet varieties on a regular basis may play an important role in the prevention and dietary management of oxidative stress-associated diseases. When comparing with the reported antioxidant properties of commonly consumed cereals including rice, wheat, corn, barley, and oats, Ravi, Rawana, and Oshadha finger millet varieties possess high antioxidant properties. These findings act as a useful guide in selecting foods for daily consumption and are helpful in decision-making for the food industry to take the advantages of finger millet flour as an alternative or supplement to other cereal flours.

Collectively, the findings of the present study expanded the current knowledge on bioactive properties of the finger millet varieties which are currently cultivated and consumed in Sri Lanka and highlighted their therapeutic properties. Further studies will be conducted to identify the specific phenolic compounds which are responsible for each activity.

## Figures and Tables

**Table 1:** Total phenolic content (TPC) and total flavonoid content (TFC) of ethanolic and methanolic extracts of Sri Lankan finger millet varieties.

Extract	TPC	TFC
Ravi E	421.15 ± 5.84^d^	108.62 ± 0.32^b^
Rawana E	288.81 ± 1.90^f^	98.74 ± 2.10^c^
Oshadha E	528.02 ± 3.72^b^	98.78 ± 0.35^c^
Ravi M	507.40 ± 4.38^c^	113.72 ± 0.89^a^
Rawana M	380.48 ± 4.36^e^	107.50 ± 0.49^b^
Oshadha M	652.11 ± 3.26^a^	107.11 ± 0.42^b^

Results are presented as mean ± SE (*n* = 6) on a dry weight basis. TPC is given as mg gallic acid equivalents per 100 g of flour. TFC is given as mg quercetin equivalents per 100 g of flour. Within a column, mean values superscripted with different letters are significantly different at *P* < 0.05 while mean values superscripted with the same letter are not significantly different at *P* ≥ 0.05. E: ethanolic extract; M: methanolic extract.

**Table 2:** DPPH and ABTS cation radical scavenging activities of ethanolic and methanolic extracts of Sri Lankan finger millet varieties.

Extract/standard	DPPH radical scavenging activity	ABTS cation radical scavenging activity
Ravi E	133.99 ± 1.54^c^	25.54 ± 0.69^b^
Rawana E	283.31 ± 4.96^a^	49.62 ± 1.13^a^
Oshadha E	231.76 ± 2.15^b^	17.34 ± 0.17^c^
Ravi M	70.48 ± 1.78^e^	11.56 ± 0.15^d^
Rawana M	117.58 ± 0.67^d^	25.13 ± 0.29^b^
Oshadha M	62.06 ± 0.58^e^	15.07 ± 0.18^c^
Trolox	7.45 ± 0.02^f^	5.71 ± 0.07^e^

Results are presented as mean ± SE (*n* = 6). All scavenging activities are given as IC_50_ values (*μ*g/ml). Within a column, mean values superscripted with different letters are significantly different at *P* < 0.05 while mean values superscripted with the same letter are not significantly different at *P* ≥ 0.05. E: ethanolic extract; M: methanolic extract.

**Table 3:** Oxygen radical absorbance capacity (ORAC), ferrous ion chelating (FIC) activity, and ferric reducing antioxidant power (FRAP) of ethanolic and methanolic extracts of Sri Lankan finger millet varieties.

Extract	ORAC	FIC activity	FRAP
Ravi E	942.48 ± 15.96^b^	22.42 ± 0.14^e^	354.46 ± 4.96^d^
Rawana E	937.42 ± 17.93^b^	15.83 ± 0.10^f^	349.52 ± 2.18^d^
Oshadha E	1160.42 ± 14.39^a^	22.63 ± 0.18^d^	554.97 ± 5.94^b^
Ravi M	971.27 ± 10.65^b^	76.63 ± 0.31^a^	495.31 ± 1.27^c^
Rawana M	784.13 ± 12.79^c^	69.94 ± 0.30^b^	501.37 ± 4.53^c^
Oshadha M	1181.62 ± 17.13^a^	68.37 ± 0.24^c^	830.76 ± 8.97^a^

Results are presented as mean ± SE (*n* = 6). ORAC and FRAP values are given as mg trolox equivalents per 100 g of flour on a dry weight basis. FIC activities are given as chelation % at 5 mg/ml assay concentration. Within a column, mean values superscripted with different letters are significantly different at *P* < 0.05 while mean values superscripted with the same letter are not significantly different at *P* ≥ 0.05. E: ethanolic extract; M: methanolic extract.

**Table 4:** Arachidonate-5-lipoxygenase (A5-LOX) and xanthine oxidase enzyme inhibitory activities and oxidative burst inhibitory activities of ethanolic and methanolic extracts of Sri Lankan finger millet varieties.

Extract/standard	A5-LOX inhibitory activity	Xanthine oxidase inhibitory activity	Oxidative burst inhibitory activity	
			Whole human blood	Isolated polymorphonuclear neutrophils
Ravi E	627.25 ± 7.35^b^	868.32 ± 6.64^a^	52.7 ± 1.3^b^	1.56 ± 0.14^b^
Rawana E	684.10 ± 7.52^a^	892.69 ± 5.61^a^	59.3 ± 0.9^a^	1.70 ± 0.12^b^
Oshadha E	638.70 ± 8.98^b^	832.57 ± 3.84^b^	59.1 ± 0.8^a^	1.35 ± 0.19^b^
Ravi M	587.97 ± 4.76^c^	781.95 ± 2.62^cd^	26.9 ± 0.6^c^	0.29 ± 0.02^c^
Rawana M	590.08 ± 6.53^c^	797.20 ± 5.90^c^	27.4 ± 0.3^c^	0.47 ± 0.05^c^
Oshadha M	484.42 ± 5.54^d^	764.34 ± 8.47^d^	27.7 ± 1.0^c^	0.44 ± 0.03^c^
Baicalein	1.76 ± 0.15^e^	NA	NA	NA
Allopurinol	NA	108 ± 0.62^e^	NA	NA
Ibuprofen	NA	NA	11.18 ± 1.87^d^	2.47 ± 0.59^a^

Results are presented as mean ± SE (*n* = 6). All inhibitory activities are given as IC_50_ values (*μ*g/ml). Within a column, mean values superscripted with different letters are significantly different at *P* < 0.05 while mean values superscripted with the same letter are not significantly different at *P* ≥ 0.05. NA: not applicable; E: ethanolic extract; M: methanolic extract.

## Data Availability

Data sets of this study are available from the corresponding author upon request.

## References

[1] Soomro S. (2013). *Identification of New Natural/Synthetic Anti-Inflammatory Molecules that Modulate Reactive Oxygen and Nitrogen Species Activities, [Ph.D. thesis]*.

[2] Perera H. D. S. M., Samarasekera J. K. R. R., Handunnetti S. M., Weerasena O. V. D. S. J. (2016). In vitro anti-inflammatory and anti-oxidant activities of Sri Lankan medicinal plants. *Industrial Crops and Products*.

[3] Schneider I., Bucar F. (2005). Lipoxygenase inhibitors from natural plant sources. Part 1: medicinal plants with inhibitory activity on arachidonate 5-lipoxygenase and 5-lipoxygenase [sol] cyclooxygenase. *Phytotherapy Research*.

[4] O'Donnell S. R. (1999). Experimental and Clinical Pharmacology: Leukotrienes - biosynthesis and mechanisms of action. *Australian Prescriber*.

[5] Azmi S. M. N., Jamal P., Amid A. (2012). Xanthine oxidase inhibitory activity from potential Malaysian medicinal plant as remedies for gout. *International Food Research Journal*.

[6] Atta-ur-Rahman M., Choudhary I., Thomsen W. J. (2001). *Bioassay Techniques for Drug Development*.

[7] Girish K. S., Kemparaju K., Nagaraju S., Vishwanath B. (2009). Hyaluronidase inhibitors: a biological and therapeutic perspective. *Current Medicinal Chemistry*.

[8] Bhattacharyya A., Chattopadhyay R., Mitra S., Crowe S. E. (2014). Oxidative stress: an essential factor in the pathogenesis of gastrointestinal mucosal diseases. *Physiological Reviews*.

[9] Shaikh R. U., Pund M. M., Gacche R. N. (2016). Evaluation of anti-inflammatory activity of selected medicinal plants used in Indian traditional medication system in vitro as well as in vivo. *Journal of Traditional and Complementary Medicine*.

[10] Salim S. (2014). Oxidative stress and psychological disorders. *Current Neuropharmacology*.

[11] Lobo V., Patil A., Phatak A., Chandra N. (2010). Free radicals, antioxidants and functional foods: impact on human health. *Pharmacognosy Reviews*.

[12] Kimatu B. M., Zhao L., Biao Y. (2017). Antioxidant potential of edible mushroom (*Agaricus bisporus*) protein hydrolysates and their ultrafiltration fractions. *Food Chemistry*.

[13] Viswanath V., Urooj A., Malleshi N. G. (2009). Evaluation of antioxidant and antimicrobial properties of finger millet polyphenols ( _Eleusine coracana_ ). *Food Chemistry*.

[14] Mathanghi S. K., Sudha K. (2012). Functional and phytochemical properties of finger millet (*Eleusine Coracana* L.) for health. *International Journal of Pharmaceutical, Chemical and Biological Sciences*.

[15] Chandra D., Chandra S., Pallavi, Sharma A. K. (2016). Review of Finger millet (*Eleusine coracana* (L.) Gaertn): A power house of health benefiting nutrients. *Food Science and Human Wellness*.

[16] Kumar A., Metwal M., Kaur S. (2016). Nutraceutical value of finger millet [*Eleusine coracana* (L.) Gaertn.], and their improvement using omics approaches. *Frontiers in Plant Science*.

[17] Jayawardana S. A. S., Samarasekera J. K. R. R., Hettiarachchi G. H. C. M., Gooneratne J. (2021). Formulation and quality evaluation of finger millet (*Eleusine coracana* (L.) Gaertn.) flour incorporated biscuits. *Food Science and Technology International*.

[18] Singleton V. L., Orthofer R., Lamuela-Raventos R. M. (1999). Analysis of total phenols and other oxidation substrates and antioxidants by means of folin-ciocalteu reagent. *Methods in Enzymology*.

[19] Gursoy N., Sarikurkcu C., Cengiz M. (2009). Antioxidant activities, metal contents, total phenolics and flavonoids of seven *Morchella* species. *Food and Chemical Toxicology*.

[20] Blois M. S. (1958). Antioxidant Determinations by the Use of a Stable Free Radical. *Nature*.

[21] Re R., Pellegrini N., Proteggente A., Pannala A., Yang M., Rice-Evans C. (1999). Antioxidant activity applying an improved ABTS radical cation decolorization assay. *Free Radical Biology and Medicine*.

[22] Ou B., Hampsch-Woodill M., Prior R. L. (2001). Development and validation of an improved oxygen radical absorbance capacity assay using fluorescein as the fluorescent probe. *Journal of Agricultural and Food Chemistry*.

[23] Carter P. (1971). Spectrophotometric determination of serum iron at the submicrogram level with a new reagent (ferrozine). *Analytical Biochemistry*.

[24] Benzie I. F. F., Szeto Y. T. (1999). Total antioxidant capacity of teas by the ferric reducing antioxidant power assay. *Journal of Agricultural and Food Chemistry*.

[25] Tappel A. L. (1962). *Methods in Enzymology*.

[26] Lee S. K., Mbwambo Z. H., Chung H. (1998). Evaluation of the antioxidant potential of natural products. *Combinatorial Chemistry & High Throughput Screening*.

[27] Sahasrabud A., Deodhar M. (2010). Anti-hyaluronidase, Anti-elastase Activity of *Garcinia indica*. *International Journal of Botany*.

[28] Haklar G., Sayin-Özveri E., Yüksel M., Aktan A. Ö., Yalçin A. S. (2001). Different kinds of reactive oxygen and nitrogen species were detected in colon and breast tumors. *Cancer Letters*.

[29] Helfand S. L., Werkmeister J., Roder J. C. (1982). Chemiluminescence response of human natural killer cells. I. The relationship between target cell binding, chemiluminescence and cytolysis. *Journal of Experimental Medicine*.

[30] Abeysekera W. K. S. M., Jayawardana S. A. S., Abeysekera W. P. K. M., Yathursan S., Premakumara G. A. S., Ranasinghe P. (2017). Antioxidant potential of selected whole grain cereals consumed by Sri Lankans: a comparative *in vitro* study. *Sri Lankan Journal of Biology*.

[31] Almeida M. M. B., de Sousa P. H. M., Arriaga A. M. C. (2011). Bioactive compounds and antioxidant activity of fresh exotic fruits from northeastern Brazil. *Food Research International*.

[32] Suriano S., Iannucci A., Codianni P. (2018). Phenolic acids profile, nutritional and phytochemical compounds, antioxidant properties in colored barley grown in southern Italy. *Food Research International*.

[33] Valko M., Rhodes C. J., Moncol J., Izakovic M., Mazur M. (2006). Free radicals, metals and antioxidants in oxidative stress-induced cancer. *Chemico-Biological Interactions*.

[34] Rahman K. (2007). Studies on free radicals, antioxidants, and co-factors. *Clinical Interventions in Aging*.

[35] Ebrahimzadeh M. I., Pourmorad F., Bekhradnia A. R. (2018). Iron chelating activity, phenol and flavonoid content of some medicinal plants from Iran. *African Journal of Biotechnology*.

[36] Jabeen A. (2013). *Study of the Suppression of Inflammatory Arthritis at Molecular Level Bb Natural and Synthetic Inhibitors of TNF-α and IL-1β, [Ph.D. thesis]*.

[37] Wisastra R., Dekker F. J. (2014). Inflammation, cancer and oxidative lipoxygenase activity are intimately linked. *Cancers*.

[38] Kostić D. A., Dimitrijević D. S., Stojanović G. S., Palić I. R., Đorđević A. S., Ickovski J. D. (2015). Xanthine Oxidase: Isolation, Assays of Activity, and Inhibition. *Journal of Chemistry*.

